# NH_4_^+ ^directed assembly of zinc oxide micro-tubes from nanoflakes

**DOI:** 10.1186/1556-276X-6-491

**Published:** 2011-08-11

**Authors:** Weiyi Yang, Qi Li, Shian Gao, Jian Ku Shang

**Affiliations:** 1 Materials Center for Water Purification, Shenyang National Laboratory for Materials Science, Institute of Metal Research, Chinese Academy of Sciences, Shenyang, 110016, People's Republic of China; 2 Department of Materials Science and Engineering, University of Illinois at Urbana-Champaign, Urbana, IL 61801, USA

**Keywords:** ZnO micro-tubes, nanoparticles, NH_4_^+ ^directed growth, self-assembly

## Abstract

A simple precipitation process followed with the heat treatment was developed to synthesize ZnO micro-tubes by self-assembly of nanoflakes composed of nanoparticles. The resulting ZnO micro-tubes demonstrated excellent photocatalytic performance in degrading methylene blue (MB) under UV illumination. It was found that NH_4_^+ ^ion played a critical role in directing the assembly of the nanoflakes to form the micro-tube structure. A critical reaction ratio existed at or above which the ZnO micro-tubes could be obtained. For the mixtures of solutions of (NH_4_)_2_CO_3 _and zinc salt, the ratio () was 2:1.

## Introduction

The zinc oxide (ZnO) has been widely investigated and utilized in various technical fields, including pigments, rubber additives, gas sensors, varistors, semiconductors, optoelectronic devices, light-emitting diodes, and solar cells, due to its catalytic, electrical, optoelectronic, and photochemical properties [1]. With the development of nanotechnology, nano/micro-sized ZnO had attracted extensive research attentions over the past decade [2-30]. Abundant nanostructure morphologies exist for ZnO, such as flower-like nanostructures [5,26,30], nanorod [3,12-15,21], nanowires [4,18], nanobridges and nanonails [17], tubular microstructural [7], nano/micro-sized particles [9,11,27,28], and micro-tubes [19]. A variety of methods had been developed to synthesize various ZnO nanostructures, including chemical vapor transport and condensation (CVTC) [23], electrodeposition [24], hydrothermal synthesis [25,26], evaporation formation [27], chemical precipitation [28], and aqueous solution deposition [29]. For example, nanohelixes, nanosprings, nanorings, and nanobelts had been synthesized by Kong and Wang via a solid-vapor process in 2003, which could have applications as one-dimensional nanoscale sensors, transducers, and resonators [20]. In 2006, Wang and Song synthesized ZnO nanowires array by the vapor-liquid-solid process, which has the potential of converting mechanical, vibrational, and/or hydraulic energy into electricity for powering nanodevices [21].

In this work, a simple precipitation process followed with the heat treatment was developed to synthesize ZnO micro-tube structure by self-assembly of nanoflakes composed of nanoparticles. The formation mechanism of this interesting ZnO morphology was examined by systematically investigating the effects from zinc salt type, precipitation agent concentration, precipitation environment, and precipitation agent type. The study identified a key role played by NH_4_^+ ^ion in the directional growth of the micro-tube structure. A critical reactant ratio () was found at 2.0:1.0, below which no such micro-tube structure could be obtained. The photocatalytic performance of ZnO micro-tubes was demonstrated by their good photocatalytic degradation effect on MB under UV illumination. With the combination of the special catalytic, electrical, optoelectronic, and photochemical properties of ZnO and this interesting highly porous micro-tube structure, these ZnO micro-tubes may find potential applications in many technical areas.

## Experimental section

### Materials

Zinc acetate dihydrate (Zn(CH_3_COO)_2_·2H_2_O, ≥99.0%, Sinopharm Chemical Reagent Co., Ltd., Shanghai, People's Republic of China) and zinc sulfate heptahydrate (ZnSO_4_·7H_2_O, ≥99.5%, Kemiou Chemicals Co. Ltd., Shenyang, People's Republic of China) were used as the zinc source, and ammonium carbonate ((NH_4_)_2_CO_3_, NH_3_% ≥40.0%, Sinopharm Chemical Reagent Co., Ltd.) and sodium carbonate (Na_2_CO_3_, ≥99.8%, Sinopharm Chemical Reagent Co., Ltd.) were used as the precipitation reagents in the synthesis of self-assembled ZnO micro-tubes, respectively. Methylene blue trihydrate (C_16_H_18_ClN_3_S·3H_2_O, Kemiou Chemicals Co. Ltd.) was used as the model organic pollutant for the static photocatalytic degradation experiment with ZnO micro-tubes under UV irradiation. All the reagents were of analytical grade and used as received without further purification.

### Synthesis

ZnO micro-tubes were synthesized by a simple precipitation method. In a typical synthesis process, a metal alkoxide, Zn(CH_3_COO)_2_·2H_2_O, was dissolved in deionized (DI) water to obtain solution #1 at the concentration of 1 M, and (NH_4_)_2_CO_3 _was dissolved in DI water to obtain solution #2 at the concentration of 1.8 M. While the mixture was stirred vigorously during the precipitation process, 100 mL of solution #1 was dropwise added into 200 mL of solution #2. After the addition of solution #1, the mixture was kept stirring for 30 min, and then the white precipitate was collected by centrifugation, washed with DI water repeatedly until neutral pH, and dried at 60°C to approximately 70°C for a day. The final ZnO product was obtained by calcination of the precipitate at 300°C for 2 h in air. To examine the effect of zinc salt on the morphology of obtained ZnO, an inorganic zinc salt, ZnSO_4_·7H_2_O, was also used in this synthesis processes with the same experimental setting as Zn(CH_3_COO)_2_·2H_2_O. To examine the precipitation reagent concentration effect on the formation of ZnO micro-tubes, (NH_4_)_2_CO_3 _solutions with different concentrations (from 1.8 to 0.5 M) were prepared and used in the precipitation process to obtain desired  ratios. The chemical addition sequence in the precipitation process was examined with both zinc salts at the  ratio of 3.2:1.0 to demonstrate the precipitation environment effect, in which both the addition of the zinc salt solution into the (NH_4_)_2_CO_3 _solution and the addition of the (NH_4_)_2_CO_3 _solution into the zinc salt solution were adopted. Na_2_CO_3 _was also used as the precipitation reagent to verify the effect of NH_4_^+ ^in the formation of ZnO micro-tubes at the  ratio of 3.2:1.0 for both zinc salts under the same experimental conditions.

### Characterization

The crystal structures of the precipitates and ZnO final products were analyzed by the D/MAX-2004-X-ray powder diffractometer (Rigaku Corporation, Tokyo, Japan) with Ni-filtered Cu (0.15418 nm) radiation at 56 kV and 182 mA. Field emission scanning electron microscopy (FESEM) and transmission electron microscopy (TEM) were utilized to study their morphologies. SEM images were obtained with a SUPRA35 Field Emission Scanning Electron Microscope (Carl Zeiss NTS GmbH, Carl-Zeiss-Straße 56, 73447 Oberkochen, Germany). SEM samples were made by dispersing the precipitate or ZnO final product in ethanol, applying drops of the dispersion on a conductive carbon tape, and drying in air for 12 h. Before imaging, the sample was sputtered with gold for 120 s (Emitech K575 Sputter Coater, Emitech Ltd., Ashford Kent, UK). TEM observation was carried out on a JEOL 2010 transmission electron microscope (JEOL Ltd., Tokyo, Japan) operated at 200 kV, with point-to-point resolution of 0.28 nm. TEM samples were made by dispersing the precipitate or ZnO final product on a Cu grid. The UV-vis spectrum of ZnO micro-tubes was measured on a UV-2550 spectrophotometer (Shimadzu Corporation, Kyoto, Japan).

### Photocatalytic degradation of methylene blue

The photocatalytic performance of ZnO micro-tubes was examined by their photodegradation of MB under UV irradiation. The initial concentration of MB aqueous solution is 1.46 × 10^5 ^mol/L (approximately 4.67 ppm) and a fixed concentration of 1 mg photocatalyst per milliliter. The average intensity of UV (254 nm) irradiance striking the MB solution was ca. 1.52 mW/cm^2^, measured by a Multi-Sense UV-B UV radiometer (Beijing Normal University Photoelectricity Instruments Plant, Beijing, China). The UV irradiation time varied from 20 to 180 min. At each time interval, ZnO micro-tubes were recovered by centrifugation at 12,600 rpm, and the light absorption of the clear solution was measured by the UV-2550 spectrophotometer. The remaining concentration of MB in the solution could be calculated by the ratio between the light absorptions of photocatalyst-treated and untreated MB solutions. For the comparison purpose, the concentration changes of MB solution were also investigated with the same experimental setup in the absence of ZnO micro-tubes and under UV light illumination, or with the presence of ZnO micro-tubes and no UV illumination.

## Results and discussion

### ZnO micro-tubes by self-assembled nanoparticles

**Figure 1:**
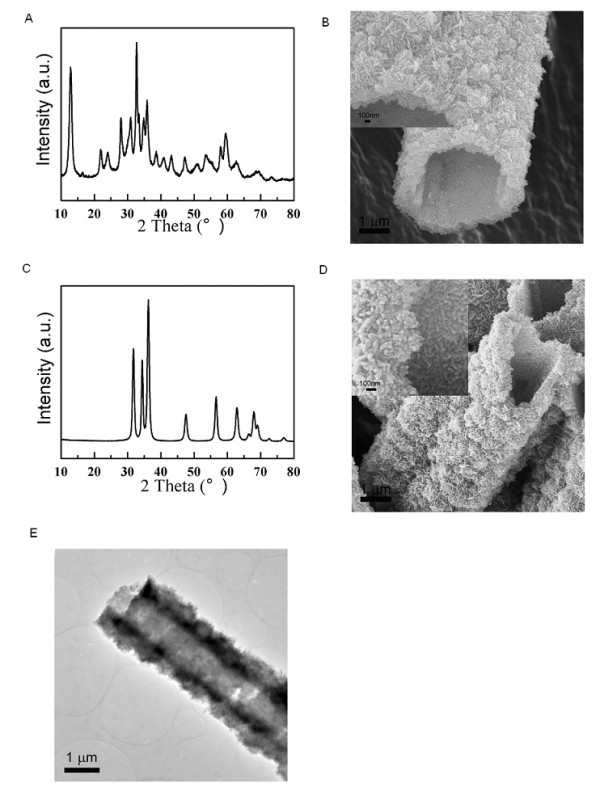
**X-ray diffraction pattern, FESEM, and TEM images**. (**A**) The X-ray diffraction pattern and (**B**) FESEM image of the white precipitate after the precipitation reaction between Zn(CH_3_COO)_2_·2H_2_O and (NH_4_)_2_CO_3 _with a molar ratio at 1.0:3.6. (**C**) The X-ray diffraction pattern, (**D**) FESEM image, and (**E**) TEM image of ZnO micro-tubes after the heat treatment of the precipitate in (A).

Figure 1A shows the X-ray diffraction pattern of the white precipitate after the precipitation reaction between Zn(CH_3_COO)_2_·2H_2_O and (NH_4_)_2_CO_3 _with a molar ratio at 1.0:3.6, which demonstrates that the precipitate obtained by the precipitation reaction is crystallized Zn_4_CO_3_(OH)_6_·H_2_O. The reaction could be expressed by:(1)

The white Zn_4_CO_3_(OH)_6_·H_2_O precipitate demonstrates an interesting tube morphology at micrometer size, which is assembled by nanoflakes composed of nanoparticles (Figure 1B). These micro-tubes have a tri-pore structure, in which the largest pores are the tubes at micrometer size, the middle ones are the inter-nanoflake pores, and the smallest ones are the pores between nanoparticles in the nanoflakes.

To convert the white Zn_4_CO_3_(OH)_6_·H_2_O precipitate to ZnO, a heat treatment was conducted at 300°C for 2 h in air. Figure 1C shows the X-ray diffraction pattern of the white precipitate after the heat treatment, which matches well to the standard diffraction pattern of wurtzite ZnO. The average crystallite size of the hexagonal phase is approximately 13.4 nm, obtained by the Scherrer's formula [31]:(2)

Interestingly, the white ZnO final product has the similar micro-tube morphology as that of Zn_4_CO_3_(OH)_6_·H_2_O. Figure 1D, E shows the FESEM and TEM images of ZnO with different magnifications. From these observations, it is clear that the micro-tube morphology was kept during the heat treatment, while the diameter of these micro-tubes became smaller due to the contraction during the heat treatment. Thus, an interesting micro-tube structure for ZnO could be obtained by a simple precipitation process followed with the heat treatment, which has a highly porous structure and could find potential applications in many technical areas.

### Effect of the type of zinc salt on ZnO structure morphology

**Figure 2:**
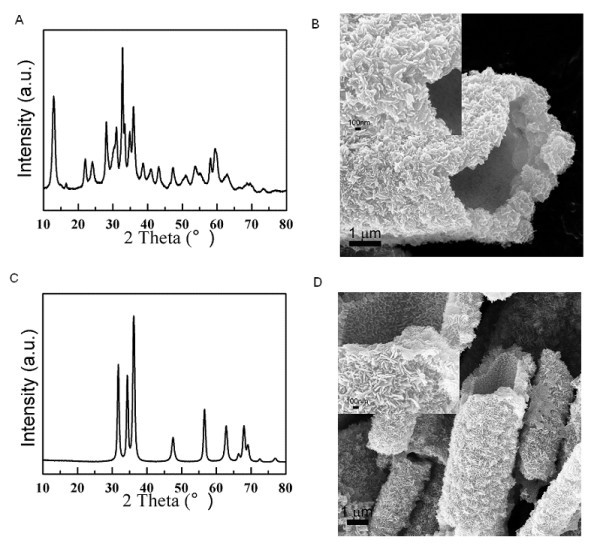
**X-ray diffraction pattern and FESEM images**. (**A**) The X-ray diffraction pattern and (**B**) FESEM image of the white precipitate after the precipitation reaction between ZnSO_4_·7H_2_O and (NH_4_)_2_CO_3 _with a molar ratio at 1.0:3.6. (**C**) The X-ray diffraction pattern and (**D**) FESEM image of ZnO micro-tubes after the heat treatment of the precipitate in (A).

To investigate the formation mechanism of this interesting micro-tube structure by the assembly of nanoflakes composed of nanoparticles, the zinc salt type effect was first examined. As a metal alkoxide, the acetate ions from Zn(CH_3_COO)_2_·2H_2_O used in the precipitation process may contribute to the formation of this micro-tube structure. To clarify its role in this process, an inorganic zinc salt, ZnSO_4_·7H_2_O, was chosen to synthesize ZnO under the same experimental conditions. Figure 2A shows the X-ray diffraction pattern of the white precipitate after the precipitation reaction between ZnSO_4_·7H_2_O and (NH_4_)_2_CO_3 _with a molar ratio at 1.0:3.6, which demonstrates that the precipitate obtained by the precipitation reaction is also crystallized Zn_4_CO_3_(OH)_6_·H_2_O. The reaction could be expressed by:(3)

The white Zn_4_CO_3_(OH)_6_·H_2_O precipitate obtained from ZnSO_4_·7H_2_O also demonstrates the similar tube morphology at micrometer size assembled by nanoflakes composed of nanoparticles (Figure 2B). After the heat treatment, similar highly crystallized ZnO micro-tubes were also obtained (Figure 2C, D), although no acetate ions were involved in this synthesis process. No obvious difference was observed on the crystal structure and morphology of the obtained ZnO final product. Thus, the type of zinc salts (organic or inorganic) is not the determining factor on the formation of ZnO micro-tubes.

### Precipitation reagent concentration effect on ZnO structure morphology

**Figure 3:**
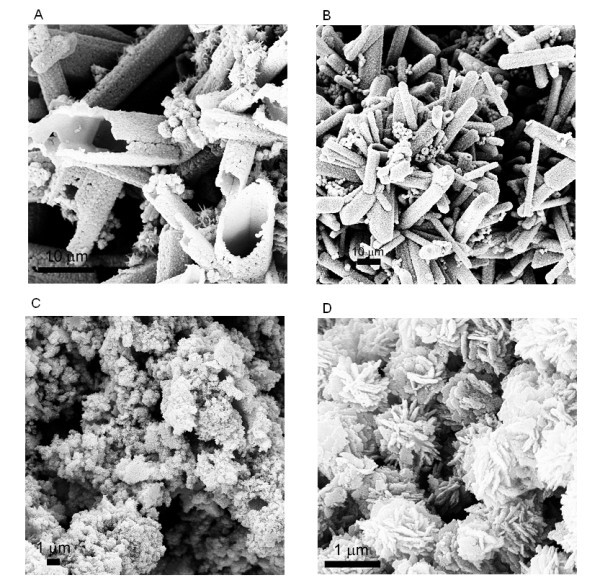
**FESEM images of ZnO nanostructures**. Obtained from the precipitation reaction between Zn(CH_3_COO)_2_·2H_2_O and (NH_4_)_2_CO_3 _with the  ratio at (**A**) 2.4:1.0, (**B**) 2.0:1.0, (**C**) 1.6:1.0, and (**D**) 1.0:1.0.

**Figure 4:**
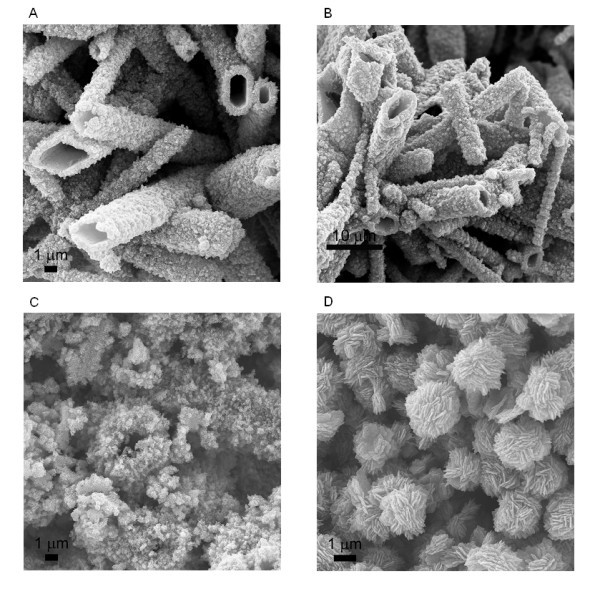
**FESEM images of ZnO nanostructures**. Obtained from the precipitation reaction between ZnSO_4_·7H_2_O and (NH_4_)_2_CO_3 _with the  ratio at (**A**) 2.4:1.0, (**B**) 2.0:1.0, (**C**) 1.6:1.0, and (**D**) 1.0:1.0.

From the above analysis, the precipitation reagent used in our experiment, (NH_4_)_2_CO_3_, should be the determinative factor in the formation of ZnO micro-tubes. To clearly demonstrate its effect, the morphology evolution of ZnO was investigated with the decrease of (NH_4_)_2_CO_3 _to Zn(CH_3_COO)_2_·2H_2_O/ZnSO_4_·7H_2_O molar ratio in the precipitation reaction, and the results were summarized in Table 1. From Table 1, a critical  ratio exists at approximately 2.0:1.0 for the use of either Zn(CH_3_COO)_2_·2H_2_O or ZnSO_4_·7H_2_O. When the  ratio is at or over 2.0:1.0 (up to 3.6:1.0 in current work), ZnO exhibited this interesting micro-tube structure. Below this critical ratio, no micro-tube structure could be obtained. Irregular agglomerated ZnO nanoparticles were obtained when  was 1.6:1.0 or 1.2:1.0. When the  ratio was 1.0:1.0, ZnO exhibited a sphere-like structure composed of nanoflakes similar to what Wang and Muhammed reported before [26]. Representative FESEM images of these ZnO structures are shown in Figure 3 (with Zn(CH_3_COO)_2_·2H_2_O) and Figure 4 (with ZnSO_4_·7H_2_O) with the  ratio at 2.4:1.0, 2.0:1.0, 1.6:1.0, and 1.0:1.0, respectively, which clearly demonstrated the ZnO structural evolution with the decrease of  ratio.

**Table 1:** The evolution of the morphology with the two zinc salts

	Zn(CH_3_COO)_2_·2H_2_O	ZnSO_4_·7H_2_O
3.6:1.0	Micro-tubes	Micro-tubes
3.2:1.0	Micro-tubes	Micro-tubes
2.8:1.0	Micro-tubes	Micro-tubes
2.4:1.0	Micro-tubes	Micro-tubes
2.0:1.0	Micro-tubes	Micro-tubes
1.6:1.0	Irregular agglomerated particles	Irregular agglomerated particles
1.2:1.0	Irregular agglomerated particles	Irregular agglomerated particles
1.0:1.0	Sphere-like microstructures consisted of nanoflakes	Sphere-like microstructures consisted of nanoflakes

### Effect of the precipitation environment on ZnO structure morphology

**Figure 5:**
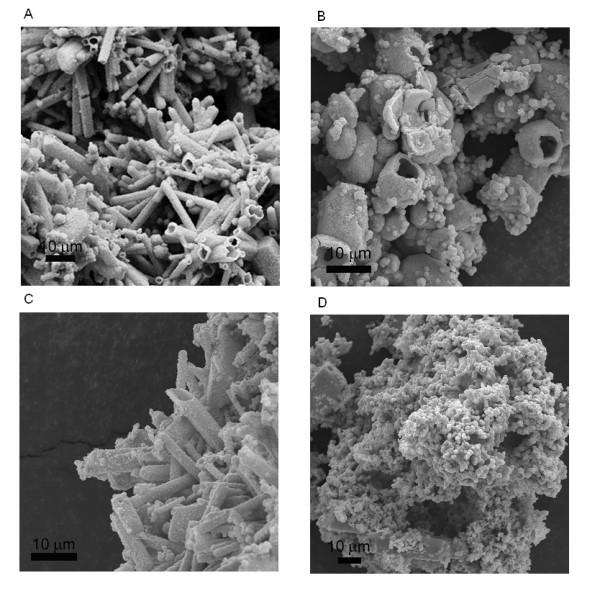
**The FESEM images of ZnO nanostructures obtained at the  ratio of 3.2:1.0**. (**A**) Zn(CH_3_COO)_2_·2H_2_O solution was added into (NH_4_)_2_CO_3 _solution, and (**B**) (NH_4_)_2_CO_3 _solution was added into the Zn(CH_3_COO)_2_·2H_2_O solution. (**C**) ZnSO_4_·7H_2_O solution was added into (NH_4_)_2_CO_3 _solution, and (**D**) (NH_4_)_2_CO_3 _solution was added into ZnSO_4_·7H_2_O solution.

To further explore the formation mechanism of ZnO micro-tubes, the effect of chemical addition sequence in the precipitation process was examined. Figure 5A shows the FESEM image of ZnO structure obtained at the  ratio of 3.2:1.0 when the addition of the Zn(CH_3_COO)_2_·2H_2_O solution into the (NH_4_)_2_CO_3 _solution was adopted in the precipitation process. ZnO micro-tubes self-assembled by ZnO nanoparticles were obtained. However, when the addition of the (NH_4_)_2_CO_3 _solution into the Zn(CH_3_COO)_2_·2H_2_O solution was adopted in the precipitation process, no micro-tube structures were obtained even with the same  ratio of 3.2:1.0 (Figure 5B). Similar result was observed with the use of ZnSO_4_·7H_2_O in this process as demonstrated in Figure 5C, D. Thus, ZnO micro-structure could not be obtained without a (NH_4_)_2_CO_3_-rich environment, no matter which zinc salt was used in the precipitation process.

### Effect of the ammonium existence on ZnO structure morphology

**Figure 6:**
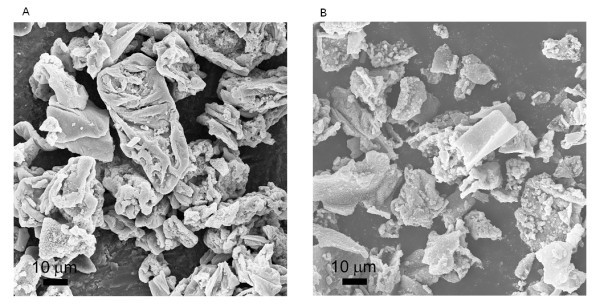
**FESEM images of ZnO nanostructures obtained with the  ratio of 3.2:1.0**. From the precipitation reaction between (**A**) Zn(CH_3_COO)_2_·2H_2_O and Na_2_CO_3_, and (**B**) ZnSO_4_·7H_2_O and Na_2_CO_3_.

Another precipitation agent, Na_2_CO_3_, was used to further examine the formation mechanism of ZnO micro-tubes in our study. Figure 6A shows the FESEM image of ZnO structure obtained at the  ratio of 3.2:1.0. The addition of the Zn(CH_3_COO)_2_·2H_2_O solution into the Na_2_CO_3 _solution was adopted in the precipitation process, which provides a Na_2_CO_3_-rich environment. From Figure 6A, irregular agglomerated ZnO nanoparticles were obtained under such experimental conditions, and no micro-tube structure was obtained. Similar result was observed with the use of ZnSO_4_·7H_2_O in this process as demonstrated in Figure 6B. Thus, ZnO micro-tubes could be obtained with (NH_4_)_2_CO_3 _as the precipitation reagent with proper  ratios, while a similar carbonate precipitation reagent Na_2_CO_3 _could not produce ZnO micro-tubes.

In the precipitation process, CO_3_^2- ^ion is one of the key components to produce Zn_4_CO_3_(OH)_6_·H_2_O precipitate, which could then be converted to ZnO by the heat treatment. To form the micro-tube structure, however, CO_3_^2- ^ion shows little effect. The experimental result here suggests that NH_4_^+ ^ion is the key factor in the formation of this micro-tube structure. Otherwise, the usage of Na_2_CO_3 _as the precipitation agent should also result in the formation of micro-tube structure as (NH_4_)_2_CO_3 _did. Thus, a possible mechanism could be proposed for the formation of these micro-tubes assembled by nanoflakes composed of nanoparticles based on the above experiment results. In the precipitation process, large amounts of NH_4_^+ ^ions exist in the reaction mixture, which do not chemically participate in the formation of the Zn_4_CO_3_(OH)_6_·H_2_O precipitate. As suggested by Wang and Muhammed [28], NH_4_^+ ^ions could adsorb onto Zn_4_CO_3_(OH)_6_·H_2_O nanoparticles just precipitated from the reaction mixture, form a monolayer on the surface of these nanoparticles, and hold the nanoparticles together by H-bonding. In their work, they observed that rod-shaped particles consisting of several spherical particles aligned in one direction. Here, the interaction between NH_4_^+^-coated Zn_4_CO_3_(OH)_6_·H_2_O nanoparticles form nanoflakes first, and the interaction between NH_4_^+^-coated Zn_4_CO_3_(OH)_6_·H_2_O nanoflakes bonds the nanoflakes together in one direction and produce micro-tube structures by self-assembly. This proposed mechanism could explain the huge difference observed on the precipitate morphology by the chemical addition sequence. When the Zn(CH_3_COO)_2_·2H_2_O solution was dropwise added into the (NH_4_)_2_CO_3 _solution, plenteous NH_4_^+ ^ions existed that could adsorb onto Zn_4_CO_3_(OH)_6_·H_2_O precipitate to cover its surface and direct the formation of micro-tube morphology. When the (NH_4_)_2_CO_3 _solution was dropwise added into the Zn(CH_3_COO)_2_·2H_2_O solution, however, not enough NH_4_^+ ^ions existed that could adsorb onto Zn_4_CO_3_(OH)_6_·H_2_O precipitate to cover its surface. Thus, the directional growth of Zn_4_CO_3_(OH)_6_·H_2_O was not achievable and no micro-tube structure was obtained.

### Light absorbance property and photocatalytic performance of ZnO micro-tubes

**Figure 7:**
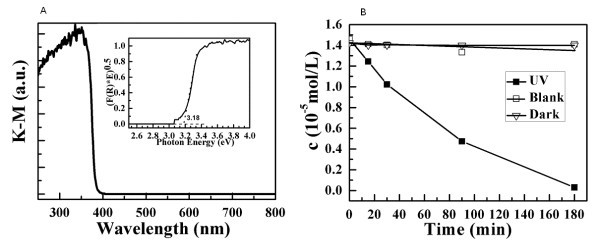
**Optical absorbance spectrum and residue MB concentration**. (**A**) The optical absorbance spectrum (in term of Kubelka-Munk equivalent absorbance units) of ZnO micro-tubes. (Note that the insert image demonstrates the Tauc Plot constructed from (A), and the band gap value is determined from the extrapolation of the linear Tauc Region line to the photon energy abscissa.) (**B**) The residue MB concentration as a function of treatment time for three different treatments: UV light illumination only (empty square), with ZnO micro-tubes but no UV illumination (empty inverted triangle), and with ZnO micro-tubes under UV irradiation (solid square).

The optical property of ZnO micro-tubes was investigated by measuring their diffuse reflectance spectra. From the reflectance data, optical absorbance can be approximated by the Kubelka-Munk function, as given by Equation 4:(4)

where *R *is the diffuse reflectance [32]. Figure 7A shows the optical absorbance spectrum of ZnO micro-tubes, which demonstrates that these ZnO micro-tubes have a strong absorption when light wavelength is < 400 nm. The insert image in Figure 7A shows the Tauc Plot [32] ((*F*(*R*)*hv)*^n ^*vs *hv*) constructed from Figure 7A in order to determine the band gap of ZnO micro-tubes. As a direct band gap semiconductor, *n *equals 0.5 for ZnO. Extrapolation of this line to the photon energy axis yields the semiconductor band gap of these ZnO micro-tubes at 3.18 eV, which is slightly smaller than the band gap of ZnO powders at 3.37 eV. The red-shift of the light absorption of these ZnO micro-tubes may be attributed to their special micro-tube morphology. Similar observation had been reported on TiO_2 _with a nanotube morphology [33].

The light absorption spectrum suggests that these ZnO micro-tubes may have a good photocatalytic performance under UV irradiation. The photocatalytic activity of these ZnO micro-tubes was investigated by its degradation effect on MB under UV irradiation. Figure 7B summarizes the residue MB concentration as a function of treatment time for three different treatments. When MB solution was under UV illumination without the addition of ZnO micro-tubes, no significant degradation could be observed. With the addition of ZnO micro-tubes, significant degradation still could not be observed when there was no UV illumination. This observation suggests that adsorption of MB will not contribute much to its concentration changes during the photocatalytic degradation treatment. Under UV light illumination, however, photodegradation of MB was clearly observed with the treatment of ZnO micro-tubes. After 3 h of treatment under UV illumination, the color of the MB solution changed from blue to almost colorless, and the concentration of residue MB was determined to near zero. From the comparison of these three treatments, it is clear that these ZnO micro-tubes have a good photocatalytic activity under UV illumination.

## Conclusions

ZnO micro-tube structure was synthesized by a simple precipitation process followed with heat treatment. The micro-tube was formed by self-assembly of nanoflakes of ZnO nanoparticles, creating a highly porous structure. The formation mechanism of ZnO micro-tube structure was investigated, and the key role of NH_4_^+ ^ion in the directional growth of this micro-tube structure was demonstrated. A critical reactant ratio () was found at 2.0:1.0, below which no such micro-tube structure could be obtained. These ZnO micro-tubes demonstrated a good photocatalytic degradation effect on MB under UV illumination and could find potential applications in many technical areas.

## Competing interests

The authors declare that they have no competing interests.

## Authors' contributions

WY carried out the synthesis, characterization, and phtocatalytic degradation experiments, and participated in the preparation of the manuscript. QL conceived of the study, participated in its design and coordination, and wrote the manuscript. SG participated in the synthesis experiment. JKS participated in the design of the study and the preparation of the manuscript. All authors read and approved the final manuscript.
